# Safety and Efficacy of Cryoballoon Ablation of Atrial Fibrillation in relation to the Patients' Age: Results from a Large Real-World Multicenter Observational Project

**DOI:** 10.1155/2021/9996047

**Published:** 2021-12-28

**Authors:** Luigi Sciarra, Saverio Iacopino, Giuseppe Arena, Claudio Tondo, Paolo Pieragnoli, Giulio Molon, Massimiliano Manfrin, Antonio Curnis, Antonio Dello Russo, Giovanni Rovaris, Giuseppe Stabile, Leonardo Calò, Gabriele Boscolo, Roberto Verlato

**Affiliations:** ^1^Cardiology Department, Policlinico Casilino, Rome, Italy; ^2^Maria Cecilia Hospital, GVM Care&Research, Cotignola (RA), Italy; ^3^Nuovo Ospedale Delle Apuane, Massa, Italy; ^4^Heart Rhythm Center at Monzino Cardiac Center, IRCC Dept. of Clinical Sciences and Community Health, University of Milan, Milan, Italy; ^5^Careggi Hospital, Florence, Italy; ^6^IRCCS Sacro Cuore Don Calabria Hospital, Negrar, Verona, Italy; ^7^Ospedale Centrale di Bolzano, Divisione di Cardiologia, Bolzano, Italy; ^8^Spedali Civili, Brescia, Italy; ^9^Cardiology and Arrhythmology Clinic, Biomedical Science and Public Health Department, Polytechnic University, Ancona, Italy; ^10^Ospedale San Gerardo, Azienda Socio Sanitaria Territoriale, Monza, Italy; ^11^Clinica Montevergine Mercogliano (AV), Casa di Cura San Michele, Maddaloni (CE), Italy; ^12^Azienda ULSS 3 Ospedale di Chioggia, Chioggia, Italy; ^13^ULSS 6 Euganea, Camposampiero, Italy

## Abstract

**Background:**

The real-world efficacy and safety of atrial fibrillation (AF) ablation in particularly young and elderly patients are still under debate. The aim of the analysis was to investigate the effect of age on the efficacy and safety of cryoballoon ablation (CBA).

**Methods:**

2,534 patients underwent pulmonary vein isolation (PVI) by way of CBA for paroxysmal or persistent drug-resistant and symptomatic AF. The population was divided into age quartiles for evaluation, including (1) <53 years, (2) ≥53 and <61 years, (3) ≥61 and <67 years, and (4) ≥67 years. Furthermore, outcomes were analyzed in patients <41 years, ≥41 and ≤74, and >74 years old. Procedural data and complications were collected, and atrial fibrillation recurrences were evaluated during follow-up.

**Results:**

Procedural-related complications (4.1%) were similar in the four subgroups according to age. At the 12-month follow-up, freedom from AF recurrence was 79.2%, 77.4%, 76.8%, and 75.2% (*p*=0.21), respectively (with increasing age). At 24-month follow-up, similar incidences of AF recurrence were observed in the four subgroups. When the sample was arbitrarily divided into the three age groups, a higher rate of recurrence was observed in older patients with regard to long-term follow-up (freedom from AF recurrence was 71.8% and 40.9%, respectively, at 12 and 24-month follow-up). In the univariate and multivariate analysis, age did not result in a significant predictor of AF recurrence during follow-up; however, a trend toward higher AF recurrences rates in patients ≥67 years was observed.

**Conclusion:**

The data demonstrated a high degree of safety during CBA across all patient ages. Procedural performance and complications were similar between different ages; AF recurrences seem to be more frequent in patients over 74 years.

## 1. Introduction

Atrial fibrillation (AF) is the most common sustained arrhythmia encountered in clinical practice [1]. The prevalence of arrhythmia is about 2% in the general population, and it significantly increases with age [1–4]. It has been observed that AF prevalence is between 10% and 17% in people aged 80 years or older [5]. In view of the increased life expectancy in the developed world, AF in the elderly becomes a significant public health problem [4, 5]. Moreover, AF has been correlated to increased morbidity and mortality, particularly in older patients [4, 5].

Catheter ablation has emerged as a cornerstone in the treatment of atrial arrhythmia with a good benefit-to-risk ratio, particularly in patients with drug-refractory symptomatic AF [6–13]. To date, many ablative schemes have been proposed, but strategies that target the pulmonary veins (PVs) are the cornerstone for most ablative procedures [14–16]. In order to simplify ablative techniques for pulmonary vein isolation (PVI), several “one-shot” devices have been proposed. Among these novel devices, cryoballoon ablation (CBA) of PVs has been widely used, and the efficacy and safety have been comparable to point-by-point radiofrequency ablation (RFA), in prospective randomized trials [17, 18]. Therefore, for the first time, the ESC guidelines on AF management published in 2016 indicated PVI by RFA or CBA without preference [19].

The efficacy and safety of catheter ablation of AF in the elderly population have not been deeply investigated, and older patients are often not represented in clinical trials, due in part to age-based inclusion and exclusion criteria. However, such a population could particularly benefit from AF ablation from a theoretical point of view. Conversely, older patients are more likely to have comorbidities that could increase the risks of ablative procedures. Additionally, the benefit of catheter ablation in terms of safety and efficacy in young adults has not been demonstrated in a large cohort study. Consequently, the aim of this analysis was to investigate the safety and efficacy of CBA among patients with AF in a large population collected in a multicenter real-world project. Specifically, this analysis evaluated quartiles of patient age during a CBA procedure using a PVI strategy of ablation with the specific aim of examining the potential differences in response for the young and old patients with regard to efficacy and safety.

## 2. Methods

From April 2012 to September 2018, consecutive patients suffering from recurrent, symptomatic, and drug-refractory AF underwent an index PVI procedure with the cryoballoon (Arctic Front or Arctic Front Advance; Medtronic, Inc.) in 47 Italian centers participating in the One-Shot TO Pulmonary vein isolation (1STOP) ClinicalService® project. Exclusion criteria were as follows: (1) permanent AF, (2) previous catheter ablation of AF, (3) New York Heart Association functional class IV, (4) unstable angina or acute myocardial infarction within three months, (5) need for or prior cardiac surgery within six months, and (6) contraindication to treatment with oral anticoagulants. Patients were prospectively followed up according to each center's clinical practice through standard in-hospital visits, remote monitoring reports, and/or telephonic visits. ClinicalService® is a national cardiovascular data repository and medical care project designed to describe and improve the quality of diagnostic and therapeutic strategies using technologies and therapies in the Italian clinical practice [20, 21]. A charter assigns the ownership of data to the participating centers and governs the conduct and relationship of the scientific committee and Medtronic. During this project, Medtronic did not have any role in identifying research objectives, interpreting results, or drafting the original manuscript. This project was approved by each site's Institutional Review Board and Local Ethics Committees. The design conforms to the principles outlined in the 1975 Declaration of Helsinki as reflected in the *a priori* approval by the institution's human research committee. Each patient included in the ClinicalService® project provided informed consent for the data collection and analysis.

The objective of this research was to assess whether procedural times, procedure-related complications, and AF recurrences differed according to the patients' age group when evaluated in quartiles. Quartiles have been defined according to the years of age of the study population (Supplementary Figure 1). In summary, the primary safety endpoint was procedural-related complications that occurred during the catheter ablation procedure or after the ablative procedure. The primary efficacy endpoint was the recurrence of AF, defined as the detection of AF both symptomatic and asymptomatic (at least 30 sec in duration when assessed with ECG monitoring) after a landmark 90-day blanking period. Before reviewing the data, the physician committee predefined the primary safety and efficacy endpoints, and the physician committee chose to divide the population into four groups according to the statistical distribution of quartiles in age at the index ablation, including (1) <53 years, (2) ≥53 and < 61 years, (3) ≥61 and <67 years, and (4) ≥67 years.

### 2.1. Subanalysis (Three Age Group Analysis)

Also, the committee decided to present the efficacy and safety outcomes in patients <41 years (defined as “very young” patients group) and in patients >74 (defined as “very old” patients group). This second grouping of patients (Supplementary Figure 1) was arbitrary and conducted in order to examine data from a cohort of patients that are underrepresented in the published literature.

### 2.2. Ablative Procedure

The CBA procedure has been previously described in detail [22, 23]. Each center utilized its own standard-of-care practices and approaches during the cryoablation procedure. In general, subjects were treated under general anesthesia or conscious sedation. A transseptal needle puncture for left atrial access was immediately followed by a heparin bolus delivery, and the subsequent heparin delivery was administered while monitoring the activated clotting time. Most often, a purpose-built dedicated delivery sheath (FlexCath, Medtronic, Inc.) was used to advance the balloon catheter and guidewire assembly during the ablation procedure. CBA procedures were performed with a 23 mm and/or 28 mm cryoballoon, which was delivered by an over-the-wire method into the left atrium. The number of freeze applications and the length of individual freezes were determined by the hospital standard-of-care usage. In addition, postablation testing methods were left to the discretion of the physician operator; however, acute PVI was the intraprocedural efficacy endpoint, which was consistently defined as electrical conduction isolation confirmed by bidirectional block. In general, acute PVI was assessed using the dedicated balloon inner-lumen diagnostic mapping catheter (Achieve mapping catheter, Medtronic, Inc.) and/or a lasso style circular diagnostic mapping catheter.

### 2.3. Data Collection and Follow-Up

Routine follow-up assessments were conducted in accordance with the standard of care and clinical practice of the participating centers by means of hospital visits and/or telephone interviews. Follow-up visits were scheduled for every 3 months after the index CBA procedure during the first 12-month period of follow-up (after the index ablation procedure). Each follow-up examination included an AF-related symptoms review, electrocardiography (ECG) for arrhythmic event assessment, and Holter monitoring according to the clinical practice. Patients were asked to provide any additional Holter or ECG records since the previous visit. The management of antiarrhythmic drugs (AADs) was left to the clinical practice of each center. After the 12-month visit, subject follow-up was performed every 6 months. If subjects missed their scheduled follow-up visit, they or their relatives were contacted by telephone. After two unsuccessful attempts at phone contact, information on the patient's life status was collected from the National Office of Vital Statistics (Italy). The first 90 days after AF ablation were denoted as the “landmark” blanking period during which no efficacy endpoint failures were calculated to allow for postablation healing without penalty to the efficacy endpoint assessment [24]. Thereafter, during the efficacy follow-up assessment, recurrence of atrial arrhythmia was defined as the detection of AF (at least 30 seconds in duration by ECG monitoring) after the performance of a single CBA procedure, with or without the use of AADs. All reported procedural-related complications were recorded, and adverse event classifications of minor or major events were made in accordance with previously published worldwide surveys on AF ablation.

### 2.4. Statistical Analysis

Descriptive statistics were used to summarize patient characteristics. These data include mean, standard deviation, minimum, maximum, and median with the interquartile range for continuous variables. Categorical variables were described by counts and percentages. Summary statistics were reported with a maximum of two decimals, as appropriate. Comparisons between groups have been performed using Kruskal Wallis's Test for continuous variables, while comparisons of categorical variables have been performed by means of the Chi-square test. Statistical tests were based on a two-sided significance level of 0.05. The analyses of time-to-the-first event were described by means of Kaplan-Meier curves and compared between the groups (two-by-two) by means of the adjusted Log-Rank Test. The follow-up duration (in months) has been computed from the date of the index ablation procedure to the date of the last available follow-up or the date of the efficacy failure event. The annual rates of complications were reported, together with the 95% Poisson Confidence Intervals. The Poisson regression model was used to calculate the incidence rate ratio (IRR), with the d-scale option. An IRR <1 would show a higher incidence of event in the reference group, while an IRR >1 would show a lower incidence of event in the reference group. Cox regression was used in both univariate and multivariate analyses to detect predictors for AF recurrences. Parameters that were significant from the univariate analysis (*p* < 0.10) were analyzed in a multivariate model, with a stepwise selection. Variables were kept in the model if they were significant (*p* < 0.05). The Cox model prediction performance was assessed using discrimination, and the C-index was reported.

The SAS software, version 9.4 (SAS Institute Inc., Cary, NC, USA), was used to perform statistical analyses.

## 3. Results

### 3.1. Baseline Characteristic

**Figure 1:**
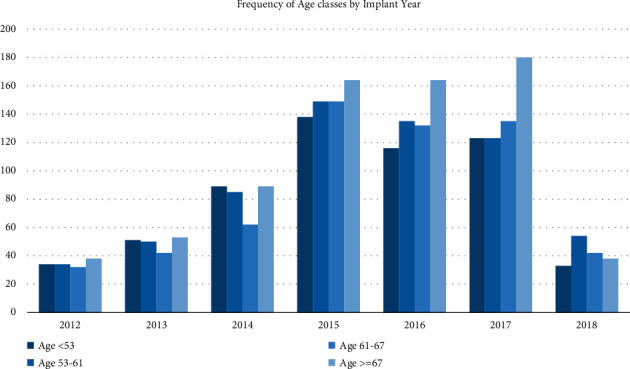
Quartiles of age distribution over enrolling years are presented. A trend toward wider indications in patients ≥67 years between 2015 and 2017 can be appreciated. However, no statistically significant differences were found in terms of age distribution (*p*=0.567). Data of 2018 were not analyzed, since the database freeze was performed in September 2018.

During this 1STOP analysis, a total of 2,534 patients underwent an index PVI by CBA. Baseline characteristics of the patient population are summarized in Table 1, and the total sample was divided into four groups according to the statistical distribution by quartiles (of age) at the time of index CBA. Trends about quartiles of age distribution over enrolling years are presented in Figure 1.

In the three age groups subanalysis, the study population has been arbitrarily divided into three clinically relevant groups, including the “very young” patient group (131 patients; age ≤40 years), intermediate age group (2,281 patients; age >40 and <75 years), and “very old” patients (122 patients; age ≥75 years).

### 3.2. Clinical Outcomes: Procedural Data and Complications

Procedural data and complications are summarized in Tables 2 and 3. Within the quartile analyses (Table 2), procedural duration, fluoroscopy time exposure, and left atrial dwell time were comparable between the four cohorts with no statistical difference between groups. Only 2.3% of the patients were treated using 2.3 mm cryoballoon according to the operator's judgment. The percentage of successfully isolated PVs was slightly but significantly lower in the younger group when compared to the other three groups (*p* < 0.001). Complication rates were comparable in the four groups studied.

### 3.3. Clinical Outcomes: AF Recurrences in Follow-Up

**Figure 2:**
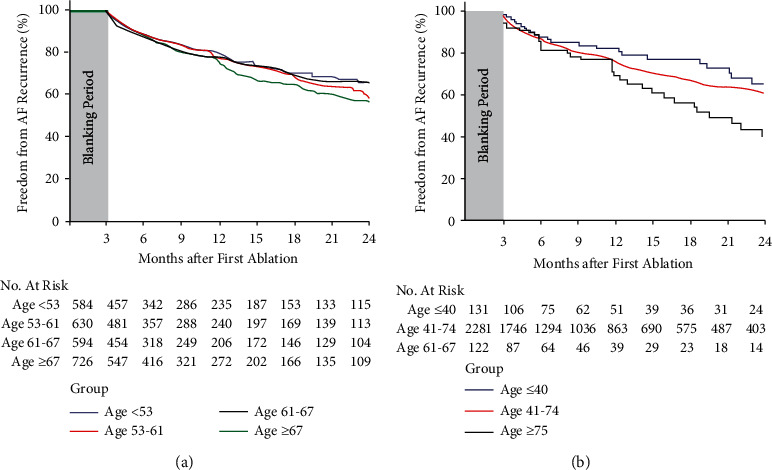
Freedom from AF recurrence in the 1STOP population. (a) Freedom from AF recurrence analyzed in four separate quartiles according to age. Overall adjusted *p* value from Log-Rank Test = 1.000. The mean follow-up duration was 15.3 ± 14.7 in the whole population with no significant difference among the groups (16.1 ± 14.9, 15.6 ± 15.2, 14.9 ± 15.0, 14.8 ± 13.9 in the four groups from the youngest to the oldest). (b) Freedom from AF recurrence analyzed in three groups according to age (age ≤ 40, 41 < age < 74, age ≥ 75). Overall adjusted *p* value from Log-Rank Test = 0.113. The mean follow-up duration in the three age's group was 16.2 ± 15.3, 15.3 ± 14.7, and 13.7 ± 14.5 in the age ≤ 40, 41 < age < 74, and age ≥ 75 groups, respectively (*p*=1.000).

The mean follow-up of the cohort of patients was 15.3 ± 14.7 months. The minimum follow-up duration was 3 months, and the maximum follow-up duration was 67.1 months. No significant differences were observed between different age groups. During follow-up, 613 (24.2%) patients had at least one AF recurrence episode. The analysis of the four subgroups (dividing population according to the statistical distribution of quartiles in age at index ablation) did not show any significant difference in terms of AF recurrences (Figure 2). In particular, the annual rate of AF recurrence was 17.5% (95% CI: 14.8%–20.7%), 17.9% (95%CI: 15.2%–21.1%), 17.69% (95%CI: 14.9%–21.0%), and 21.04 (95% CI: 18.2%–24.3%) in the four groups increasing in age, respectively. Also, the unadjusted survival analysis for the freedom from AF recurrence (Figure 2 panel a) showed no statistical difference between the groups. The 1-year survival probability was 79.2 ± 3.5%, 77.4 ± 4.3%, 76.8 ± 4.7%, and 75.2 ± 4.6% in the four quartiles, respectively (*p*=0.390). During the follow-up, 153 patients (6.0%) underwent a repeat procedure, including 50 (8.6%) in the age <53 group, 46 (7.3%) in the 53–61 age group, 28 (4.7%) in the 61–67 age group, and 29 (4.0%) in the age ≥67 group (*p*=0.009).

In univariate (Table 4) and multivariate analysis, many baseline predictors were tested to find a possible correlation with AF recurrences in follow-up. Baseline characteristics resulting in differences between groups were tested, and no significant correlation between age groups and AF recurrence was found. Even in the multivariate analysis, there was no correlation between age and AF recurrences. However, a nonstatistically significant trend toward higher AF recurrences rates in patients ≥67 years was observed. In the multivariate model, persistent AF and CHA₂DS₂-VASc resulted to be independent predictors of AF recurrences (see Table 4).

### 3.4. Three Age Groups Subanalysis

When analyzed by the three clinically relevant age groups (Table 3), there were no differences in all procedural parameters. The procedural-related complications were similar between all groups.

When considering the analysis dividing patients into three groups (very young, intermediate age, and very old patients), an increased rate of AF recurrences was observed in patients over 74 years. The 1-year freedom from AF recurrence probability was 83.1% in patients <41 years, 77.0% in the intermediate age patients, and 71.8% in patients with more than 74 years, *p* = 0.038 (Figure 2, panel b). Twelve patients (9.2%) in younger, 135 (5.9%) in the intermediate and 6 (4.9%) in the older patient groups underwent redo procedure (*p*=0.826).

## 4. Discussion

In this current evaluation of the 1STOP analysis, a population of 2,534 patients with AF who were treated by CBA using a PVI strategy of ablation were examined across quartiles of age, including (1) <53 years, (2) ≥53 and < 61 years, (3) ≥61 and < 67 years, and (4) ≥ 67 years. Ten percent of the study population were only mildly symptomatic, and ablation was indicated on the basis of a clinical evaluation, according to the clinical practice of the center and taking into account the AF burden. Among the four age groups, there were no statistical differences with regard to acute procedural parameters or procedure-related complications, which were relatively low at 4.1%. At the 12-month follow-up, freedom from AF recurrence was 79.2%, 77.4%, 76.8%, and 75.2% (*p*=0.21), respectively (with increasing age). Age did not result in a significant predictor of AF recurrence during follow-up; however, a trend toward higher AF recurrences rates in patients ≥67 years was observed.

### 4.1. AF Ablation in Young and Old Patients

The efficacy and safety of AF ablation in younger and older populations have not been deeply investigated. Despite the increasing experience of operators and significant advancements in technological support, safety is still a remarkable issue for AF ablation [25–27]. Moreover, low rates of complications are hardly acceptable in relation to the treatment of an arrhythmia, which is generally considered a “non-life-threatening” illness (particularly in very young patients). On the other hand, the worldwide population is constantly aging, and the prevalence of AF is higher in the elderly [1–5]. Therefore, many elderly patients could theoretically be good candidates for AF ablation. Although AF is associated with aging and cardiopulmonary pathologies, it is possible to observe it in young subjects, without structural heart disease. This patient subset lacks solid data on the efficacy and safety of the ablative procedures with both RFA and cryoablation. However, encouraging data have come from small sample studies showing positive feedback on recurrence of AF and complication rates of ablative procedures in the young, regardless of the ablation catheter [28–31]. Generally speaking, large studies exploring the safety and efficacy of AF ablation according to patients' age are still lacking.

The high number of comorbidities in the elderly strongly limits the use of antiarrhythmic therapy. Therefore, catheter ablation could represent a definitive solution for maintaining sinus rhythm in the elderly. However, the limited experiences available in the literature cannot scientifically support the wide use of ablation in the elderly. Some studies have explored the effects of RFA in the elderly [32–34]. Even if those studies are not homogeneous for the number of patients included, types of arrhythmia, distribution, and classes of age, an overall evaluation of those papers seems to suggest similar rates of success and complications between older and younger patients [32, 33]. In a small single-center retrospective analysis conducted on patients ≥75 years undergoing RFA, Metzner and colleagues [35] reported an incidence of 5.8% for major and 19% for minor complications. In the same report, the authors showed that after a single ablative procedure only 38% of patients were in stable rhythm after a mean follow-up of 37 ± 20 months (46% of paroxysmal AF patients, 31% of persistent AF patients, and 10% of long-standing AF patients).

CBA emerged as a promising strategy to cure AF with high procedural success rates, high durability of PVI, [36, 37], and good short- and long-term success rates with an acceptable incidence of complications in both paroxysmal and persistent AF [38–40]. Also, the Fire and Ice trial provided evidence for a noninferiority efficacy of cryoballoon versus RFA for PVI in patients with paroxysmal AF [18]. Nevertheless, patients over 75 years were not included in the trial [40]. More recently, Heeger and colleagues (in a multicenter study) compared the effects of cryoballoon ablation in 104 patients ≥75 years and in 104 propensity score-matched patients <75 years [41]. The authors reported comparable safety, short-term efficacy, and long-term efficacy in both groups. However, procedure-related major complications were reported in 6.7% of patients of both groups. Furthermore, in another study, no statistically significant differences in complication and recurrence rates were found by comparing a group of patients aged ≥75 with a group of patients aged <75 both treated with PVI by CBA for paroxysmal or persistent AF [42]. Other single-center or multicenter experiences provide further evidence for the safety and efficacy of CBA in elderly patients [43–47]. In summary, although positive and encouraging data on efficacy and safety in particularly old patients exist, they are limited and mainly based on small samples. Our analysis (including 2,534 patients) could represent an important overview highlighting CBA efficacy and safety according to the patent's age and confirming high safety and efficacy levels both in the elderly and in young patients.

### 4.2. Influence of Age on AF Cryoablation Efficacy

Our data, coming from a large real-world multicenter prospective project, seems to suggest similar outcomes of CBA in the four groups of patients, with no statistically significant differences in terms of AF recurrences between the quartiles. This observation was further confirmed by the univariate and multivariate analysis. Particularly, age did not result in being a significant predictor of AF recurrence in both univariate and multivariate analyses. However, a nonstatistically significant trend toward higher AF recurrences rates in patients ≥67 years was observed. Even if this is only “a trend,” we can speculate that such a result could be easily explained by the greater degree of “atrial cardiomyopathy” as well as a cumulative greater number of patients suffering from persistent and long-standing persistent AF in the elderly. Moreover, when the sample was divided into the three groups of patients (very young, middle aged, and elderly, respectively), significant statistical differences were obtained in the AF recurrence rates. More precisely, the Kaplan-Meier curves showed a similar recurrence trend in the three groups up to 12 months after the index procedure, but after this period, there is a significant increase in the recurrence rate in patients with age greater than 74 years when compared with the other two groups. It is known that aging involves a negative electro-anatomical remodeling of the atrium, so the patients are more vulnerable to triggering and maintaining AF. However, such results, balancing risk/benefit ratio, provide further scientific evidence to encourage the wider use of CBA both in the elderly and in the young patients, with symptomatic recurrent AF.

Another interesting point to be underlined is the lower rates of repeat procedures in patients above 67 years, despite a comparable incidence of arrhythmia recurrences in the same group. This may reflect a higher threshold to indicate a repeat procedure in the elderly, in the daily practice. The high levels of safety for cryoballoon ablation even in the elderly, as demonstrated by our paper, could encourage in future wider indications for repeat procedures even in these patient groups.

### 4.3. Safety and Procedural Outcomes of Cryoablation

As already mentioned, safety may be an important issue about AF ablation even nowadays and particularly in younger or older patients. Our analysis clearly demonstrates that procedure-related complications rates were very low and similar in the four different classes of age. Therefore, the safety of CBA is not influenced by the patient's age. The analysis in very young (age ≤40 years) or very old patients (age ≥75 years) confirmed similar complication rates in comparison to the rest of the population. In the quartile analysis, a slight but significantly lower percentage of isolated PVs in younger patients was observed. In a real-world observational study, it is not so easy to explain such a result that comes from a “real-life observation”. Also, slightly superior ablative times were observed both in the younger quartile and in the quartile of patients between 53 and 61 years. However, the quartile comparison showed no other statistically significant differences in procedural data. The possibility of offering an equal procedural performance to the fragile oldest patients or younger patients could represent a significant benefit.

## 5. Conclusion

Our large and multicenter real-world prospective registry showed high safety levels for CBA at different ages. Procedural performance indicators were similar at different ages, and complication rates were low and not related to the patient's age. Even procedural efficacy was not influenced by age. The slightly higher rates of AF recurrences should be considered in very old candidates (≥75 years), which occurred in a mid-term follow-up period.

Such results provide further scientific evidence for the use of CBA even in younger or older populations.

## 6. Limitations

We acknowledge that the median follow-up time of this analysis is 15 months and that a longer follow-up time can be needed to assess and confirm our results over time.

The percentage of the female sex is low, especially in the younger cohort of patients. This reflects the clinical practice, as per the nature of this project.

Some baseline characteristics of the study populations are different in the various groups analyzed. We believe that this limitation deals with the intrinsic nature of the registry. However, even if we do not think that those differences significantly influence the study results, they have to be taken into account.

Data on PVI durability and predominant extra PV-triggers according to patients' age were not discussed in this analysis.

## Figures and Tables

**Table 1:** Baseline patient characteristics (*N* = 2534) by quartiles.

Baseline characteristics	Total cohort (*N* = 2534)	Age <53 (*N* = 584)	Age ≥53 and <61 (*N* = 630)	Age ≥61 and <67 (*N* = 594)	Age ≥67 (*N* = 726)	*p* value
Age at first ablation (years)	59.7 ± 10.5	44.8 ± 6.7	56.7 ± 2.3	63.6 ± 1.7	71.1 ± 3.5	<0.001^1,2,3,4,5,6^
Gender (female)	27.4% (695)	17.8% (104)	22.2% (140)	30.1% (179)	37.5% (272)	<0.0011^1,2,3,4,5,6^
Body Mass index	27.0 ± 4.1	26.8 ± 4.4	27.6 ± 4.3	26.8 ± 3.9	26.8 ± 4.0	0.005^1,4,5^
Any arrhythmia symptoms	88.9% (2253)	88.9% (519)	89.2% (562)	90.1% (535)	87.7% (637)	1.000
Type of atrial fibrillation (AF)						0.847^∗^
Paroxysmal	74.9% (1899)	79.3% (463)	74.9% (472)	72.4% (430)	73.6% (534)	
Persistent	22.6% (572)	19.0% (111)	22.5% (142)	25.1% (149)	23.4% (170)	
Long-standing persistent	2.5% (63)	1.7% (10)	2.5% (16)	2.5% (15)	3.0% (22)	
Months from first AF diagnosis	55.0 ± 106.2	45.2 ± 54.7	50.9 ± 102.3	60.6 ± 141.2	62.0 ± 107.1	0.004^3^
Failed ≥2 antiarrhythmic drugs	43.6% (1106)	32.6% (190)	42.6% (268)	49.0% (291)	49.3% (357)	<0.001^1,2,3,4,5^
New York heart association class						0.003∗^1,2,3,5^
1	78.9% (1999)	85.4% (499)	80.0% (504)	79.1% (470)	72.3% (525)	
≥2	21.1% (535)	14.6% (85)	20% (126)	20.9% (124)	27.7% (201)	
History of Stroke/TIA	4.6% (116)	2.3% (13)	4.8% (30)	4.4% (26)	6.4% (47)	0.039^1,2,3^
Cardiac insufficiency	3.9% (99)	4.0% (24)	3.7% (23)	3.4% (20)	4.2% (32)	1.000
Hypertension	47.8% (1198)	27.3% (158)	45.3% (282)	52.0% (307)	62.8% (451)	<0.001^1,2,3,4,5,6^
Any valve disease	5.4% (137)	4.2% (24)	4.3% (28)	4.1% (24)	8.4% (61)	0.003^3,5,6^
CHA₂DS₂-VASc score						<0.001∗^1,2,3,4,5,6^
0	23.3% (590)	54.0% (316)	36.2% (228)	7.8% (46)	0.0% (0)	
1	30.6% (775)	37.4% (219)	41.7% (262)	34.3% (203)	12.4% (90)	
2	24.5% (620)	6.0% (35)	17.6% (110)	37.0% (220)	35.0% (255)	
3	21.6% (549)	2.3% (14)	4.7% (29)	21.0% (125)	52.4% (381)	
Diabetes	5.4% (137)	3.7% (22)	4.8% (30)	5.5% (33)	7.2% (52)	0.376
Chronic renal failure	2.3% (58)	0.0% (0)	1.4% (9)	3.1% (18)	4.4% (31)	<0.001^1,2,3,5^
Ischemic cardiopathy	6.0% (153)	1.7% (10)	3.9% (24)	8.1% (47)	9.7% (69)	<0.001^1,2,3,4,5^
Hypertensive cardiopathy	15.8% (401)	8.2% (48)	15.1% (95)	15.0% (89)	23.3% (169)	<0.001^1,2,3,5,6^
Primitive cardiomyopathy	3.3% (84)	4.1% (24)	4.2% (27)	3.0% (18)	2.0% (15)	0.618
Left ventricular ejection fraction (%)	59.2 ± 7.0	59.1 ± 7.5	59.6 ± 6.6	59.3 ± 6.7	58.8 ± 7.2	0.570
Left atrial area (cm^2^)	22.1 ± 6.1	20.7 ± 5.0	22.2 ± 7.3	22.6 ± 5.2	22.9 ± 6.2	<0.001^1,2,3^
Antiarrhythmic drug usage	71.8% (1819)	70.0% (408)	72.5% (457)	73.9% (439)	71.0% (515)	1.000
Anticoagulant therapy usage	82.9% (2100)	72.2% (422)	82.3% (519)	86.3% (514)	88.8% (645)	0.570

^1^A significant difference between *age<53* and *age ≥53 and <61*; ^2^a significant difference between *age <53* and *age ≥61 and <67*; ^3^a significant difference between *age <53* and *age ≥67*; ^4^a significant difference between *age ≥53 and <61* and *age ≥61 and <67*; ^5^a significant difference between *age ≥53 and <61* and *age ≥67*; ^6^a significant difference between *age ≥61 and <67* and *age ≥67*. ^•^The *p* was referred to as the differences in group distribution.

**Table 2:** Procedural and safety characteristics (*N* = 2534) by age groups divided into quartiles.

Procedural characteristics	TOTAL (*n* = 2534)	Age <53 (*n* = 584)	Age 53–61 (*n* = 630)	Age 61–67 (*n* = 594)	Age ≥67 (*n* = 726)	*p* value
Procedure duration (min)	106.2 ± 46.5	110.0 ± 49.6	108.1 ± 45.8	104.0 ± 45.2	103.4 ± 45.3	0.064
Fluoroscopy time (min)	28.5 ± 27.4	28.5 ± 14.7	29.8 ± 48.0	27.8 ± 15.7	28.0 ± 15.5	1.000
Ablation time (min)	25.0 ± 17.5	26.0 ± 19.1	26.0 ± 17.2	23.9 ± 16.7	24.1 ± 17.0	0.025^4,5^
% Of isolated PVs (*n* treated veins/*n* target veins)	98.1% (9547/9729)	96.7% (2200/2269)	98.6% (2371/2404)	98.3% (2222/2261)	98.5% (2754/2795)	<0.001^1,2,3^
Left atrium dwell time (min)	57.0 ± 26.7	59.8 ± 28.5	58.1 ± 27.2	55.9 ± 26.0	54.8 ± 25.1	0.150
Patients with at least one complication	4.1% (104)	3.8% (22)	3.8% (24)	4.0% (24)	4.7% (34)	1.000
Permanent diaphragmatic paralysis	0.0% (1)	0.2% (1)	0.0% (0)	0.0% (0)	0.0% (0)	1.000
Transient diaphragmatic paralysis	1.8% (46)	1.5% (9)	2.2% (14)	1.9% (11)	1.7% (12)	1.000
Pericardial effusion	0.2% (6)	0.3% (2)	0.0% (0)	0.0% (0)	0.6% (4)	0.610
Femoral arterio-venous fistula	0.3% (7)	0.5% (3)	0.0% (0)	0.2% (1)	0.4% (3)	1.000
Cardiac tamponade	0.3% (7)	0.2% (1)	0.2% (1)	0.3% (2)	0.4% (3)	1.000
Pneumothorax/Hemothorax	0.0% (0)	0.0% (0)	0.0% (0)	0.0% (0)	0.0% (0)	–
Femoral pseudoaneurism	0.1% (3)	0.0% (0)	0.2% (1)	0.2% (1)	0.1% (1)	1.000
TIA	0.2% (3)	0.0% (0)	0.2% (1)	0.0% (0)	0.1% (2)	1.000
Hematoma	0.4% (9)	0.2% (1)	0.3% (2)	0.5% (3)	0.4% (3)	1.000
Other complication	0.9% (22)	0.9% (5)	0.8% (5)	1.0% (6)	1.0% (6)	1.000

^1^A significant difference between *age<53* and *age ≥53 and <61*; ^2^a significant difference between *age<53* and *age ≥61 and <67*; ^3^a significant difference between *age<53* and *age ≥67*; ^4^a significant difference between *age ≥53 and <61* and *age ≥61 and <67*; ^5^a significant difference between *age ≥53 and <61* and *age ≥67*; ^6^a significant difference between *age ≥61 and <67* and *ag e≥67*.

**Table 3:** Procedural and safety characteristics (*N* = 2534) by three age groups.

Procedural characteristics	Total (*n* = 2534)	“Very young” Age ≤40 (*n* = 131)	“Intermediate age” Age 41–74 (*n* = 2281)	“Very old” Age ≥75 (*n* = 122)	*p* value
Procedure duration (min)	106.2 ± 46.5	101.3 ± 43.3	107.0 ± 46.9	97.6 ± 41.3	0.124
Fluoroscopy duration (min)	28.5 ± 27.4	26.0 ± 13.3	28.8 ± 28.5	25.4 ± 12.1	0.344
Ablation time (min)	25.0 ± 17.5	23.5 ± 14.6	25.1 ± 17.8	23.9 ± 15.2	1.000
Left atrium dwell time (min)	57.0 ± 26.7	54.7 ± 23.3	57.5 ± 27.1	51.5 ± 20.4	0.622
Patients with at least one complication	4.1% (104)	2.3% (3)	4.3% (97)	3.3% (4)	1.000
Permanent diaphragmatic paralysis	0.0% (1)	0.0% (0)	0.0% (1)	0.0% (0)	–
Transient diaphragmatic paralysis	1.8% (46)	0.8% (1)	1.9% (43)	1.6% (2)	1.000
Pericardiac effusion	0.2% (6)	0.0% (0)	0.2% (5)	0.8% (1)	1.000
Femoral arterio-venous fistula	0.3% (7)	0.8% (1)	0.2% (5)	0.8% (1)	0.776
Cardiac tamponade	0.3% (7)	0.0% (0)	0.3% (7)	0.0% (0)	1.000
Pneumothorax/Hemothorax	0.0% (0)	0.0% (0)	0.0% (0)	0.0% (0)	–
Femoral pseudoaneurism	0.1% (3)	0.0% (0)	0.1% (3)	0.0% (0)	1.000
Stroke	0.0% (0)	0.0% (0)	0.0% (0)	0.0% (0)	–
TIA	0.1% (3)	0.0% (0)	0.1% (2)	0.0% (1)	1.000
Pulmonary vein stenosis	0.0% (0)	0.0% (0)	0.0% (0)	0.0% (0)	–
Hematoma	0.4% (9)	0.0% (0)	0.4% (9)	0.0% (0)	1.000
Other complication	0.9% (22)	0.8% (1)	1.0% (22)	0.0% (0)	1.000

**Table 4:** Univariate analysis to investigate AF predictors. Baseline characteristics resulting in differences between groups (*p* < 0.10) were tested and are presented.

Variable	Univariate		Multivariate	
	Hazard ratio (HR) (95% CI)	*p* value	Hazard ratio (HR) (95% CI)	*p* value
Group (age <53 vs. Others)	0.86 (0.70–1.06)	0.168		
Group (age ≥53 and < 61 vs. Others)	1.01 (0.83–1.23)	0.891		
Group (age ≥61 and < 67 vs. Others)	0.92 (0.75–1.14)	0.459		
Group (age ≥67 vs. Others)	1.19 (0.99–1.43)	0.061		
Gender (male)	0.90 (0.75–1.09)	0.272		
Body Mass index (continuous)	1.00 (0.97–1.02)	0.894		
Type of AF-persistent	1.60 (1.33–1.92)	<.001	1.38 (1.24–1.50)	<0.001
Number of tested AAD 2+	1.22 (1.02–1.46)	0.030		
NYHA (categorical)	0.86 (0.67–1.11)	0.250		
History of stroke/TIA	1.23 (0.82–1.83)	0.321		
Hypertension	1.26 (1.06–1.50)	0.008		
Any valve diseases	1.17 (0.77–1.80)	0.462		
CHA₂DS₂-VASc (continuous)	1.09 (1.02–1.17)	0.012	1.11 (1.03–1.20)	0.006
Ischemic cardiopathy	1.00 (0.69–1.45)	0.987		
Hypertensive cardiomiopathy	1.11 (0.88–1.40)	0.364		
Left atrial area (cm^2^) (continuous)	1.01 (0.99–1.04)	0.166		
Left atrial area (cm^2^) > 21	1.22 (0.94–1.57)	0.135		
Mitral regurgitation (categorical)	0.91 (0.78–1.04)	0.173		

In bold are presented variables with a *p* < 0.10 in the univariate model, which were used in the multivariate model. In the multivariate model, the only independent predictors of AF recurrence were persistent AF and CHA₂DS₂-VASc.

## Data Availability

All data are included in the Italian ClinicalService®.

## Supplementary Materials

Supplementary Materials Supplementary Figure 1: 2534 patients enrolled divided into four quartiles according to patients age. Moreover, subgroups of “younger” patients (age ≤40 years) and older patients (age >74 years) were identified.
