# Supplementary material for: Safety and Efficacy of Cryoballoon Ablation of Atrial Fibrillation in relation to the Patients' Age: Results from a Large Real-World Multicenter Observational Project
Source: Cardiol Res Pract. 2021 Dec 28;2021:9996047. doi: 10.1155/2021/9996047 (PMC8727157; doi:10.1155/2021/9996047)
Supplement: Supplementary Materials — Supplementary Figure 1: 2534 patients enrolled divided into four quartiles according to patients age. Moreover, subgroups of “younger” patients (age ≤40 years) and older patients (age >74 years) were identified. [file 9996047.f1.doc]

**Supplementary Figure**


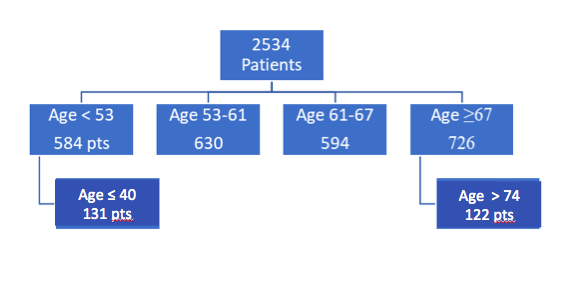


Figure Legend: 2534 patients enrolled divided into four qurtiles according to patients age. Moreove, subgroups of “younger” patients (age ≤ 40 years) and older patients (age > 74 years) were identified.
